# Digital training to support delivery of an evidence-based cardiac rehabilitation programme for heart failure: a mixed-methods evaluation of healthcare professionals’ training experiences and intervention fidelity

**DOI:** 10.1136/bmjopen-2026-122821

**Published:** 2026-10-20

**Authors:** Samantha B Van Beurden, Rosina Cross, Sinead TJ McDonagh, Carolyn Deighan, Rod S Taylor, Patrick J Doherty, Andrew Ludman, Liz Clark, Hasnain M Dalal, Colin J Greaves

**Affiliations:** 1 Health and Community Sciences, University of Exeter Medical School, Exeter, UK; 2 NHS Lothian, Edinburgh, UK; 3 School of Health and Wellbeing, University of Glasgow, Glasgow, UK; 4 Department of Health Sciences, University of York, York, UK; 5 Cardiology, Royal Devon and Exeter NHS Foundation Trust, Exeter, UK; 6 Royal Cornwall Hospitals NHS Trust, Truro, UK; 7 School of Sport, Exercise and Rehabilitation Sciences, University of Birmingham, Birmingham, UK

**Keywords:** Heart failure, Education, Medical, Quality in health care

## Abstract

**Abstract:**

**Objectives:**

The Rehabilitation Enablement in Chronic Heart Failure (REACH-HF) programme is an evidence-based, healthcare professional-facilitated, home-based cardiac rehabilitation intervention for people living with heart failure. Digital training may provide a scalable approach to preparing healthcare professionals to deliver REACH-HF. This study aimed to describe observed patterns of fidelity across REACH-HF intervention components following digital training, and to explore healthcare professional views of the training’s feasibility, acceptability and usability and its perceived influence on intervention delivery.

**Design:**

Mixed-methods implementation evaluation combining descriptive assessment of fidelity with semi-structured interviews analysed using reflexive thematic analysis.

**Setting:**

Five National Health Service cardiac rehabilitation services in England.

**Participants:**

15 healthcare professionals were recruited, 14 completed the digital training and 7 subsequently delivered REACH-HF. Fidelity data comprised 17 audio-recorded intervention sessions delivered by six facilitators. 11 healthcare professionals participated in the semi-structured interviews.

**Intervention:**

Co-designed training programme to train healthcare professionals as facilitators of the REACH-HF programme. Delivered in a hybrid format with self-paced learning modules accessible via a bespoke eLearning platform (asynchronous) with subsequent 3-hour virtual group session (synchronous) to complete the training.

**Outcome measures:**

Competence of REACH-HF delivery, assessed using an established intervention fidelity checklist, alongside qualitative inquiry concerning healthcare professional experiences of the training, including its feasibility, acceptability and usability, and its perceived influence on intervention delivery.

**Results:**

Adequate fidelity was observed across most REACH-HF components. Delivery was strongest for patient involvement, the physical activity programme and programme closure; competent delivery was observed for needs assessment, tailored planning, understanding of heart failure and progress monitoring. Lower scores were seen for emotional support, medication-related discussions and active exploration of caregiver involvement. Caregiver emotion and well-being could not meaningfully be assessed due to limited caregiver presence. Facilitators generally perceived the training as usable and valuable, but engagement and preparedness were shaped by prior experience, perceived relevance and service pressures affecting protected training time. Confidence was consolidated primarily through real-world delivery rather than training completion alone. Non-delivery reflected constrained operational opportunities rather than a consistent difference in confidence or preparedness.

**Conclusion:**

Co-designed digital training may support healthcare professional preparation for REACH-HF delivery. Further research with larger and more complete fidelity datasets is required before firm conclusions can be drawn about overall delivery fidelity or equivalence with face-to-face training. Translating digital training into practice requires protected time for training and intervention delivery, timely opportunities for delivery and ongoing practical and organisational support.

**STRENGTHS AND LIMITATIONS OF THIS STUDY**

- The mixed-methods design integrated independently scored, competency-based assessment of recorded intervention delivery with reflexive thematic analysis of facilitator interviews.
- Interviews included healthcare professionals who did and did not progress from training to delivery across five National Health Service cardiac rehabilitation services, enabling examination of this implementation transition.
- The fidelity dataset comprised only 17 recordings from six facilitator-patient pairings, with uneven recording coverage, limiting the precision and stability of item-level competence estimates. Selection, reactivity and assessor biases are possible because fidelity data came only from facilitators returning usable recordings, sessions were knowingly recorded and assessors were not formally blinded.
- The approximately 4-month mean interval between training and first delivery may have affected skill retention, but the limited fidelity dataset prevented examination of associations between delay and delivery competence.

## Introduction

 Heart failure (HF) affects an estimated 64 million people worldwide and prevalence continues to rise due to ageing populations and improved survival after cardiac events.^1^ In England alone, the number of people living with HF is expected to double by 2040 (reaching around 2 million).^2^ Cardiac rehabilitation (CR) plays a vital role in improving outcomes for people with HF and is strongly endorsed in clinical guidelines across the UK, Europe and USA.^3–5^ However, persistently low participation rates globally have prompted national health strategies to establish improvement targets, including increasing access in the USA from 20 to 70% of all eligible patients (including those with HF)^6^ and in England from 52–85% across cardiovascular disease by 2028^7^).

Meeting these targets will require scalable training and delivery models that maintain quality and person-centred practice across services. The Rehabilitation Enablement in Chronic Heart Failure (REACH-HF) programme is an evidence-based, home-based, healthcare professional (HCP)-facilitated CR intervention that can widen access by supporting delivery beyond centre-based provision and has been shown to improve health-related quality of life and reduce hospital admissions in people with HF.^8^ However, delivery of programmes such as REACH-HF depends on workforce capacity and service infrastructure, as well as commissioning and referral pathways.^9^ Delivering REACH-HF with fidelity requires HCPs to develop specific skills, which are supported through training. A process evaluation of the original REACH-HF trial found that while overall delivery quality was adequate, there was scope for improvement in more than half of recorded patient interactions,^10^ highlighting the need for more robust training.

The original REACH-HF facilitator training was delivered as a 3-day in-person course. Although effective, it was resource-intensive. In response to the COVID-19 pandemic, a 2-day remote version was rapidly implemented via videoconferencing in 2020 and has been used to train over 700 HCPs to date. However, with over 250 CR centres across the UK and high annual staff turnover,^11^ accessible and scalable training models are needed to support ongoing delivery.

Digital learning platforms are increasingly used in HCP education because they offer flexible access (including asynchronous participation), scalability and the potential for more consistent delivery of training content.^12 13^ However, an integrative review of HCP e-learning for self-management support suggests that many programmes lack interactivity and opportunities for practical application and meaningful reflection.^14^ While evidence indicates that online learning can achieve comparable outcomes to face-to-face learning, this depends on pedagogically informed design incorporating interactivity, feedback and practice.^15^ For relational interventions like REACH-HF, training therefore needs to cultivate social and teaching presence and provide repeated opportunities for applied skill development and reflection, enabling the experiential learning^16^ required to develop the interpersonal and facilitation skills to work in partnership with people living with HF. It therefore remains unclear whether digitally delivered training can support quality delivery of complex, relational CR interventions in routine service.

This study aimed to assess the quality of REACH-HF delivery following a co-designed, digitally delivered HCP facilitator training programme developed to support scalability and alongside facilitators’ perceptions of the training’s feasibility and value.

The specific objectives were to:

- Describe fidelity and variation of REACH-HF delivery following digital training.
- Explore HCP views on the feasibility, acceptability and usability of the digital training and perceived impact on intervention delivery.

## Methods

### Design

This study was a mixed-method implementation evaluation of a co-designed digital REACH-HF facilitator training course to upskill HCPs to deliver the REACH-HF intervention. The evaluation combined competency-based fidelity assessment of REACH-HF delivery using audio-recorded sessions with semi-structured interviews to explore facilitators’ experiences of the training, including feasibility (eg, integration into routine practice), acceptability, usability and perceived impact on practice. The following guidelines were used to support the transparent reporting of this study: Standards for Reporting Implementation Studies (StaRI),^17^ Standards for Reporting Qualitative Research (SRQR)^18^ and the Reflexive Thematic Analysis Reporting Guidelines (RTARG).^19^

### Participants

Fifteen (allied) HCPs from five National Health Service (NHS) CR services across England (three per site) were identified by local site leads as eligible staff who had not previously received REACH-HF training and consented to take part in training, intervention session recording and interviews.

### The digital REACH-HF facilitator training course

The digital REACH-HF facilitator training was co-designed through five iterative workshops (May 2021–March 2022) with patients living with HF, caregivers, HCPs and training providers, alongside one-to-one consultations and asynchronous feedback. Co-design activities refined intended learning outcomes and translated the existing course into a scalable digital learning experience, including interactive exercises, scenario-based videos, reflection activities, progression criteria and a live trainer-led online session. Further detail on the co-design process is provided in the online supplemental file.

The training comprised self-paced eLearning content equivalent to approximately 1.5 full-working days encouraged to be completed over approximately 1 month, followed by a 3-hour live trainer-led interactive group session delivered by two trainers (including CD) from the Heart Manual Department (NHS Lothian), the providers of the REACH-HF intervention and facilitator training, via videoconference to consolidate learning through structured skills practice, feedback, Q&A and discussion with trainers and peers. The eLearning comprised six core topic modules: (1) The REACH-HF Programme; (2) Skills for Supporting Health Behaviour Change; (3) Managing Stress, Anxiety and Low mood; (4) The REACH-HF Physical Activity and Exercise Programme; (5) Engaging Family and Friends; and (6) REACH-HF’s 7-Steps of Successful Facilitation. Although numbered, the first five modules could be accessed in any order. Completing them, including the required exercises and knowledge quizzes, unlocked a final integrative module that translated the learning into practical guidance for delivering REACH-HF and had to be completed before the live session. An additional optional module addressed other patients concerns, while downloadable slides and facilitation tools were made available to support learning and subsequent delivery. The live session consolidated learning through structured skills practice, feedback, questions and discussion with trainers and peers.

### Training delivery

The facilitator training was delivered across four cohorts between July and October 2022.

### Intervention delivery

Following completion of training, facilitators could recruit eligible patients and deliver the 12-week REACH-HF intervention. Patients with HF were eligible for REACH-HF if they:

- Were referred to the trained facilitator’s CR service.
- Had not received CR before.
- Were deemed eligible to receive CR within the service.
- Consented to enrol in REACH-HF and for their sessions to be audio-recorded.

### Data collection

We collected facilitator demographics via a brief questionnaire. To assess fidelity, facilitators audio-recorded their intervention sessions (with patient and caregiver consent, see above). With a target sample size of 15 facilitators across five sites, and anticipated up to three patients per facilitator, this was considered to provide a maximum of 45 facilitator-patient pairings and approximately 360–540 potentially recordable sessions. Based on the 34% recording availability achieved in the REACH-HF process evaluation (110/322 contacts),^10^ we anticipated approximately 122–184 recordings to describe variation across facilitators, patients and sessions. Patient level clinical outcomes were not collected, as the study was designed to assess facilitator training and fidelity of intervention delivery rather than clinical effectiveness.

We conducted semi-structured interviews with facilitators after they had completed delivery of the 12-week intervention to at least one patient. Facilitators who had not delivered REACH-HF were interviewed at the end of the study. Study data collection ended April 2023; consequently the time available to establish patient referral, recruit eligible patients and complete intervention delivery varied to training cohort and site activation date. Interviews explored experiences of the digital training course, including content, format, perceived usability and acceptability, perceived influence on facilitation practice and challenges. We developed the interview topic guide (see online supplemental file) by drawing on the REACH-HF logic model and programme theory,^20^ the facilitator training course and co-design partner feedback. Interviews were conducted via videoconference by a member of the research team (RC) between January 2023 and April 2023, audio-recorded and transcribed verbatim.

### Data analysis

Facilitator demographic data were summarised descriptively using counts and percentages for categorical variables and mean and SD for continuous variables.

#### Fidelity assessment

Fidelity was assessed by scoring the audio-recorded intervention sessions using the REACH-HF fidelity checklist (see online supplemental file) previously applied in the REACH-HF feasibility study,^20^ process evaluation of the REACH-HF trial^10^ and currently being applied in the REACH-HFpEF trial.^21^ The checklist rates enactment of key intervention components (eg, person-centred communication style, exercise support, self-care barriers, emotional support and caregiver involvement) on a 6-point competency-based rating scale informed by the Dreyfus Model of Skill Acquisition^22 23^ which conceptualises progression from novice to expert performance through increasing levels of situational understanding, flexibility and fluency in practice. Scores reflected the quality and competence of delivery of each intervention component, rather than simple presence or absence of content.

Consistent with previous REACH-HF fidelity work, a score of 3 was considered indicative of adequate delivery, representing competent enactment of the intervention component, although with some inconsistencies or areas for improvement. Lower scores reflect more limited or problematic delivery, while higher scores reflect increasingly confident, responsive and skilful enactment within the facilitator–patient interaction.^10 20^ Two members of the team (SBVB and CJG; both familiar with the REACH-HF intervention having developed the original intervention and accompanying facilitator training, and their subsequent refinements, and with expertise in fidelity assessment) independently coded a subset of audio-recorded sessions. The raters were not formally blinded to facilitator identity (eg, names mentioned within the audio-recording), site, time since training or session sequence. Scores differing by no more than 0.5 points were considered concordant (consistent with methods used by Frost *et al*^10^). Scores differing by more than 0.5 points were discussed with reference to the researchers’ item-specific qualitative notes until a consensus score was agreed. Consensus scores were used in the final fidelity analysis. Percentage agreement and quadratic weighted kappa^24^ were calculated using the original, pre-consensus, independent scores. Weighted kappa was selected because the checklist generates ordered item-level ratings, including half-point scores, and the analysis concerned agreement between two raters on these individual ratings. Quadratic weighting enabled penalising larger disagreements over smaller discrepancies. Calibration discussions identified differing interpretations of the applicability of caregiver-related items and the level at which programme closure should be assessed, leading to refinement of the scoring guidance for Items 9–12. Item 9 (caregiver involvement) was scored for all sessions even where no caregiver was present, to indicate the extent to which the facilitator was engaging in active support-network exploration to identify a potential caregiver. Items 10–11 (caregiver emotional impact and caregiver well-being) were coded as Not Applicable when no caregiver was present. Similar to previous applications of this checklist, Item 12 (programme closure) was treated as a patient-level indicator for the descriptive analysis: the highest closure score observed across a patient’s recorded sessions was assigned to all recorded sessions for that patient. For each item, session-level consensus scores were averaged within each facilitator-patient pairing. The resulting patient-level scores were then summarised across pairings using means, IQRs, (25th–75th percentiles), minima and maxima to examine variation in delivery quality. No statistical, equivalence or non-inferiority comparison with delivery following face-to-face training was planned or undertaken.

#### Semi-structured interviews

Interview transcripts were analysed using reflexive thematic analysis,^25^ treating participants’ accounts as contextually situated reflections of lived experience and acknowledging the active role of the researcher’s interpretive lens, shaped by their understanding of the intervention and practice. Analysis was primarily inductive, focusing on facilitators’ experiences of (a) the digital training course and (b) subsequent delivery of the REACH-HF intervention. Coding was led by RC with regular analytic discussion with SBVB to support reflexive engagement, refine analytic focus and consider alternative interpretations (both with expertise in qualitative health research and familiarity with the intervention^26 27^). Consistent with reflexive thematic analysis, coding was approached as an interpretive, iterative process rather than a reliability exercise, with themes developed through sustained engagement with interview data. Initial descriptive codes were generated and iteratively organised into candidate themes by RC and SBVB. These were refined through ongoing discussion and re-engagement with the full dataset to enhance coherence, distinctiveness and analytic depth. Further detail on the analytic process, including alignment with the RTARG,^19^ is provided in the online supplemental file.

### Patient and public involvement statement

Patients and caregivers with lived experience of HF were involved throughout the development and evaluation of the digitally delivered REACH-HF facilitator training. Patient and caregiver representatives were involved during funding development with the lead representative included as co-applicant. Representatives contributed through a formal patient and public involvement (PPI) advisory group, co-design workshops, recurring project meetings (lead representative only) and iterative review processes conducted via email. PPI contributors helped shape the overall study design and project priorities, refine the intended learning outcomes and review training materials, scripts and role-modelling videos to ensure communication approaches and delivery scenarios reflected lived experiences of HF and caregiving.

For the present study, patients and caregivers contributed to development and review of consent procedures associated with audio-recording of intervention delivery sessions involving patients and caregivers. PPI contributors also informed interpretation of findings, particularly regarding areas where further refinement of training and implementation support may be required, and contributed to dissemination planning and dissemination in the form of a peer-reviewed journal article (lead representative LC co-authored).

## Results

HCPs were initially recruited across five NHS CR sites. One withdrew during the online component because of long-term illness, and 14 completed both the online component and the live session of the course.

Following training completion, three facilitators did not progress to patient recruitment or intervention delivery: one became unwell (long-term illness), one was redeployed and one could not be contacted despite several attempts. Of the 11 facilitators who remained available and contactable, seven recruited patients and delivered REACH-HF across four sites, while four were unable to recruit an eligible patient within the remaining study timeframe. Governance and site-set-up delays reduced the time available for recruitment and completion of the 12-week intervention, despite an extension to the study period. In total, eight patients and one caregiver enrolled onto the REACH-HF programme. Thirty-three recordings were returned for fidelity assessment, of which 17 were suitable for coding and represented REACH-HF delivery sessions (excluding empty or inaudible recordings, false starts and practice, accidental or duplicate recordings). These comprised sessions from six facilitators, each delivering to one patient. All facilitators who returned recordings also took part in an interview. Interviews were also conducted with five other HCPs, of whom one delivered the intervention without recording it and the remainder had completed the training but had not been able to recruit a patient within the study timeframe (n=4). Collectively, the 11 interviews included facilitators from all five sites: seven who delivered the intervention and four who did not. The achieved sample was considered to provide adequate information power^28^ because the interviews focused specifically on the training and its translation into delivery and all interviewees had direct experience of the same training programme. The interviewee sample also captured variation in delivery status, professional role and service context, generating detailed accounts that supported analysis across these experiences. Figure 1 shows the flow of participants and data collection across the study. Table 1 summarises facilitator characteristics in the interviewee sample.

**Table 1:** Interview sample characteristics

	n=8*
Age (years), mean (SD)	46.3 (11.2)
Sex, n(%) female	4 (50%)
Ethnic group	
White, n(%)	6 (75%)
Non-white	2 (25%)
Highest level of education	
Postgraduate degree or higher	3 (37.5%)
Undergraduate degree or professional qualification	5 (62.5%)
Professional role	
Nurse (specialist (HF/cardiology), CR)	5 (62.5%)
Exercise specialist/physiologist	2 (25%)
Physiotherapist	1 (12.5%)

* Only eight completed demographic questionnaires were received from the 11 interviewees (six who delivered the REACH-HF intervention and two who did not). Given the small incomplete and imbalanced groups, characteristics are reported for the interview sample overall and are not compared by delivery status.

CR, cardiac rehabilitation; HF, heart failure; REACH-HF, Rehabilitation Enablement in Chronic Heart Failure.

**Figure 1:**
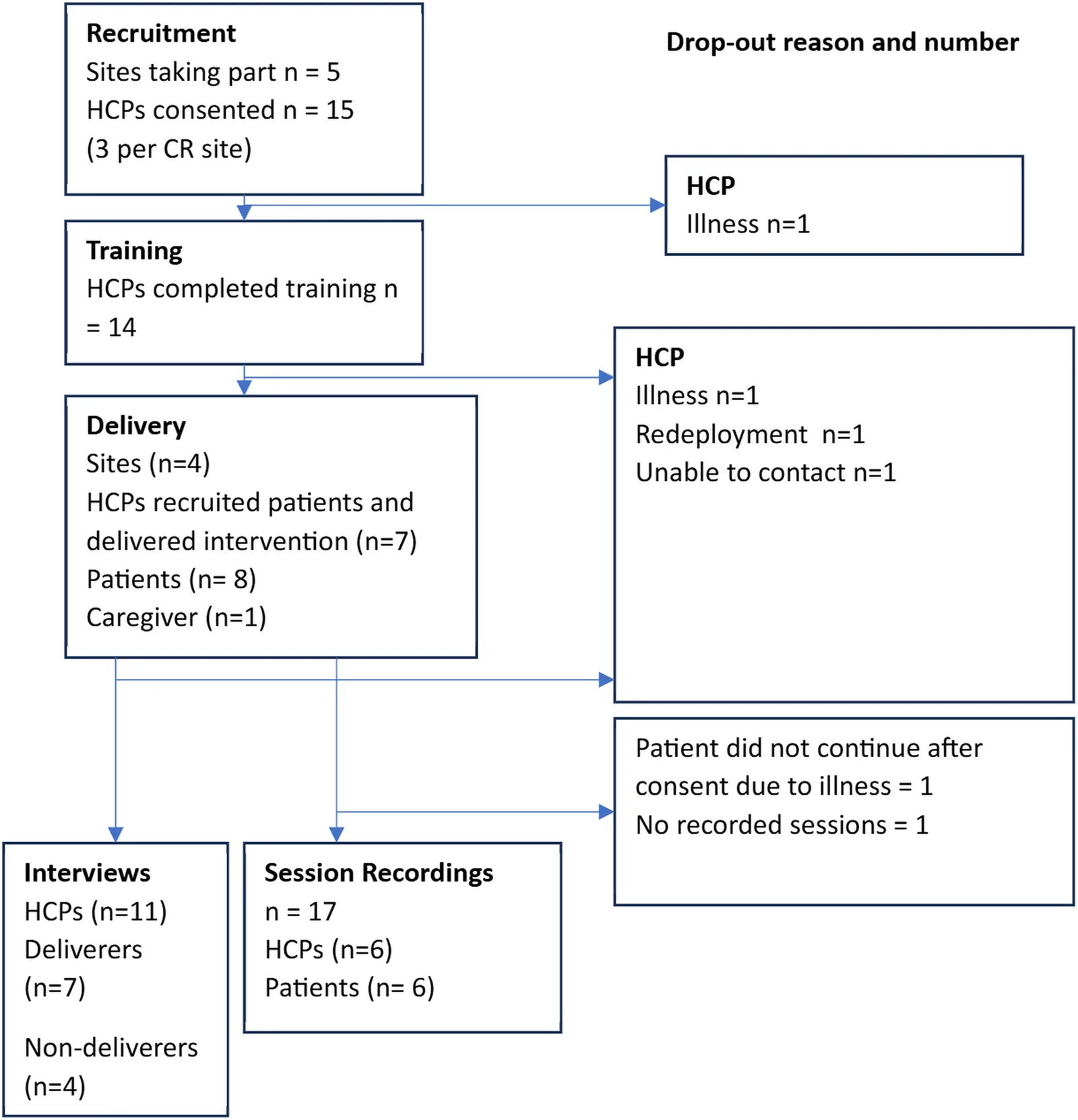
Participant flowchart. CR, cardiac rehabilitation; HCP, healthcare professional*.*

On average there was a 4-month gap between training and first-time REACH-HF intervention delivery.

Our findings are presented in two parts: (1) fidelity of intervention delivery and (2) HCPs’ perceptions of the digital training course. This is followed by an integration of the two sources of data.

### Fidelity of intervention delivery

Of the 17 suitable audio-recordings, 13 were independently scored by two researchers. Inter-rater agreement was substantial (quadratic-weighted Cohen’s κ=0.75), with 86.2% agreement on 145 comparable item ratings falling within the pre-specified tolerance of 0.5 points. Four recordings arrived late and consensus discussions had already taken place; therefore, these additional four recordings were scored by SBVB only, using the refined scoring guidance.

Mean fidelity scores indicated adequate delivery (≥3) across most aspects of REACH-HF delivery (table 2). The strongest delivery (mean>4) was observed for patient involvement (Item 1), the physical activity programme (Item 6) and programme closure (Item 12). Competent delivery (3–4) was observed for the assessment of individual needs (Item 2), tailored treatment planning (Item 3), building understanding of HF (Item 4) and progress monitoring (Items 5a and b).

**Table 2:** Fidelity of intervention delivery summary scores – scores for 17 intervention sessions, aggregated by patient level (n=6)^*^

Fidelity item	Mean	Median	IQR	Range
1 Active participant involvement	4.7	4.5	3.6–5.0	3.4–6.0
2 Assessment of individual situation and needs	3.8	3.8	3.6–4.0	3.1–4.5
3 Tailored treatment plan	3.3	3.4	2.8–3.9	2.0–4.0
4 Building understanding of HF	3.7	4.1	2.9–5.0	1.1–5.0
5.a. Support progress monitoring	3.4	3.4	2.4–4.0	2.1–5.0
5.b. Review progress	3.0	3.0	2.8–3.0	2.0–4.0
6 Physical activity plan	4.3	4.3	4.0–4.7	3.3–5.0
7 Address patient emotion	2.6	2.4	1.6–3.4	1.4–4.5
8 Medication issues	2.5	2.4	1.3–3.9	1.0–4.0
9 Encourage caregiver involvement	2.2	1.8	1.1–3.5	0.4–4.5
10 Address caregiver emotion	2.0 **^†^**	2.0^†^	NA^‡^	NA^‡^
11 Address caregiver well–being	2.0 **^†^**	2.0^†^	NA^‡^	NA^‡^
12 Preparing programme closure	4.1	4.5	3.4–4.5	3.0–5.0

* Scores from 17 recorded sessions were first aggregated within facilitator-patient pairings and subsequently summarised across pairings (n=6). Minimum and maximum scores in the present study should be interpreted with caution due to the small number of recordings per patient. (mean 2.8, range 1–8, median 1.5, IQR(Q1–Q3): 1.0–3.5). As a result, observed extremes may reflect singlesession variability rather than stable patterns of facilitator delivery across patients.

† Only one patient had a caregiver present (n=1) for scoring to be applicable for these two items.

‡ IQRs were not calculated from Items 10 and 11 because only one recorded case involved caregiver participation.

HF, heart failure; NA, not applicable.

Weaker delivery was observed for addressing patient emotion (Item 7) and medication-related discussions (Item 8). Caregiver-focused components (Items 9–11) also received lower scores overall. However, scoring for these domains was limited, as only one recorded case involved caregiver participation. Consequently, our data do not support interpretation of items caregiver emotional support and caregiver well-being (Items 10 and 11), which required a caregiver to be present. Caregiver involvement (Item 9) was scored for all sessions because it assessed whether facilitators explored and encouraged the involvement of a caregiver or other supporter, regardless of whether one was present. Its low score therefore indicates that this active exploration was inconsistently undertaken and, where evident, was often characterised by problems in delivery.

IQRs and observed ranges indicated variation in scores between facilitator-patient pairings for several intervention domains, particularly building understanding of HF, progress monitoring, addressing patient emotion, medication-related discussions and caregiver involvement (Items 4, 5a, and 7–9).

### Qualitative findings

The qualitative findings describe HCP experiences of the digital REACH-HF training and translating their learning into intervention delivery. These experiences varied across professional roles and service contexts, shaping how the training was received, understood and enacted. In line with recommended practice for reflexive thematic analysis,^25^ quotations are presented with minor edits for readability (eg, removal of disfluencies, repetitions and interviewer prompts), with care taken not to remove ambiguity, emphasis or meaning that was analytically relevant, and with pseudonyms for anonymisation.

#### Making sense of REACH-HF and my role as a provider: negotiating role fit and programme legitimacy

Facilitators described an initial period of sense-making in which they sought to understand what REACH-HF was, how it related to existing CR provision, and what it meant for their own professional role. This process was shaped by how participation in the training was initiated, how clearly expectations were communicated and whether REACH-HF was understood as a meaningful extension of current practice rather than an additional or externally imposed task. Across interviews, facilitators positioned REACH-HF in relation to what they already did, rather than as a wholly new way of working.

How facilitators were enrolled into the training influenced how they initially understood and engaged with REACH-HF. Some described being invited or encouraged to participate as part of service development or role progression, framing the training as an opportunity to extend their practice. For example, one facilitator linked their participation to planned changes within their service:

the cardiac rehabilitation team I currently work for wanted to sort of revamp their home-based programme. I was sort of gonna move to band [**] to do that sort of thing. And then, my manager thought it'd be a great idea to do this as well, so I've got experience with exercise, with people at home and stuff like that*.* Paul, delivered.

Others described participation as more usual practice, particularly where REACH-HF was introduced in team meetings as a programme the service had already decided to adopt. These different starting points shaped how facilitators initially integrated REACH-HF within their role, influencing early motivation and expectations of what participation would involve.

Facilitators consistently evaluated the programme in relation to their existing professional knowledge and service provision. Many described the training as reinforcing or validating practices they already used, rather than introducing unfamiliar concepts. One facilitator reflected positively on this alignment:

It really validated what I’d been doing anyway (…) but this is being put together in a very organised and structured way. Claire, delivered

Where training content aligned closely with existing expertise, facilitators described feeling immediately comfortable with those elements. For example, exercise specialists reported confidence in delivering exercise components based on prior experience, while identifying other aspects of the programme as more challenging:

I’m the exercise specialist (…) so I was fine in terms of delivering that. If I was new, I might have found it quite difficult. Tom, delivered.

Conversely, facilitators reported engaging more deeply with content that sat outside their usual role. Several described spending more time on components they felt less familiar with, such as exercise prescription (for non-exercise specialists) or psychological support, while moving more quickly through areas they perceived to be within their existing expertise.

There were some areas I was happier with than other (…) symptom management I was very familiar with, but the exercise I wasn’t, so I spent a bit longer on that*.* Dave, delivered.

In making sense of REACH-HF, facilitators also assessed its distinctiveness relative to existing services. Some viewed REACH-HF as filling a perceived gap between their home-based advice and group-based rehabilitation, particularly for patients who did not engage with standard provision.

I think there’s a big hole in the middle… and REACH would kind of facilitate that. Matthew, delivered.

Others experienced greater overlap with current pathways, describing REACH-HF as fitting within established routines rather than transforming them.

Whether HF rehabilitation was already embedded within local CR provision also varied. In one service, REACH-HF was introduced alongside the development of HF rehabilitation provision.

we weren’t delivering heart failure rehab prior to that at all… while REACH was coming in, we were trying to set up our own service for heart failure as well. Dave, delivered.

Elsewhere, REACH-HF had not been operationally adopted despite staff completing the training.

REACH heart failure isn’t something which on our menu of choice in our menu of choice in our heart failure service […]why do I need to be trained as well? I do get it if we’re about to launch it and it’s going to be part of our menu of choice. But it isn’t. George, did not deliver.

This facilitator described an ‘overwhelmed’ HF service in which staff activity had shifted predominantly towards HF medication management rather than rehabilitation attributed to changes in medication guidance and therefore changing patient prescriptions. By contrast, others described established referral pathways between HF and CR teams, or patients with HF already participating in existing CR provision

These early sense-making judgements about role fit, legitimacy and added value shaped not only initial engagement with REACH-HF but also how confidently facilitators engaged with the training and made decisions about what to prioritise.

#### Developing capability and confidence through the training

Facilitators described the digital training programme as central to developing their capability and confidence to deliver REACH-HF, while also highlighting practical and experiential factors that shaped how learning was taken up. Experiences of the training were not uniform. Facilitators described engaging selectively with different components depending on prior experience, available time and perceived relevance. Confidence was therefore developed differently between intervention components, with some aspects of delivery feeling well supported by the training and others remaining more tentative.

A recurring feature of facilitators’ accounts was the volume (training content itself) and pacing of the training content (shaped by their service context). Many described the programme as extensive and more time-consuming than initially anticipated, particularly where their protected time to do the training was compromised due to workforce capacity challenges (ie, case loads encroaching into ‘protected’ time). This shaped how facilitators engaged with the training, with several reporting that they frequently rescheduled their protected time for learning and eventually rushed through modules or prioritised completion over depth of engagement, to be able to attend the live session*.*

Because we’d done it in such a short space of time, it seemed like information overload almost*.* Anna, delivered.

These time pressures did not necessarily lead facilitators to view the training negatively, but they did influence the extent to which learning felt consolidated.

Facilitators described engaging selectively with different training components, often making pragmatic decisions about where to focus their attention. Written materials were generally seen as clear and accessible, while experiences of videos and reflective exercises were more mixed. Some facilitators valued videos and role-play examples as a way of visualising delivery and reinforcing learning.

I like the role play [videos)](…) it puts something in your mind to make you reflect and think. Is that something that I do? Can do? Can I use those? George, did not deliver.

Others reported skipping or fast-forwarding through videos, particularly where content felt familiar, pace was considered too slow or time was constrained.

Some of the videos were quite long-winded… I started watching and thought, actually I know where this is going. Emily, did not deliver.

These accounts suggest that facilitators actively calibrated their engagement with the training, rather than working through content in the same way.

Live training sessions complemented the digital platform by enabling facilitators to ask questions, practise skills, and learn from experienced trainers, which, alongside video materials, enhanced confidence in delivering consistent and credible patient support. Overall, despite generally positive views of the training, facilitators reported that their confidence to deliver REACH-HF after being trained was partial and component-specific. Many felt confident delivering elements aligned with their professional background, while expressing greater uncertainty around less familiar aspects. Several emphasised that, despite the usefulness of role-play videos and ability to practise in live role-playing, confidence would only fully develop through real-world delivery.

I don’t think you feel 100% confident, but I definitely felt like I had a good knowledge (…) and I think in the next few months I’ll be more confident with it*.* Anna, delivered

Technical issues within the platform, particularly early errors in quiz or completion tracking, were reported as disruptive. While these technical issues were quickly resolved through contact with the research team, facilitators described frustration when problems interrupted progression.

As a result, the training functioned less as a discrete endpoint for developing confidence, and more as a provisional foundation that facilitators expected to consolidate, adapt, and test through subsequent delivery in practice.

#### Translating training into practice: contextual constraints and experiential learning

Facilitators described the move from training to delivery as a distinct phase in which the adequacy of training was tested in practice. While the digital training was widely seen as providing a solid foundation, facilitators emphasised that confidence and capability were shaped through enactment in real-world settings, often under conditions of service pressure, variable patient engagement and limited opportunities for repetition.

Local service context became consequential during patient recruitment and delivery. Some facilitators reported that many people with HF already attended group-based CR, leaving a small pool seeking home-based provision. At one site, potentially suitable patients with HF followed separate physiotherapy pathways rather than being offered home-based CR. In another service, patients were available but staffing pressures limited delivery capacity.

[REACH-HF delivery to HF patients] requires [a] specific person and money […] it just isn’t there*.* Matthew, delivered.

At one site, prior REACH-HF training had not translated into service adoption. One facilitator reported that several colleagues had already completed REACH-HF training 2 years earlier, yet none had delivered the programme. This facilitator questioned why they were to become the next person trained when REACH-HF was not being introduced into routine provision. The other facilitator at this site referred to feeling confident to deliver the programme following the training, despite knowing prior to engaging in the training, that they would unlikely be delivering it due to lack of capacity in their caseloads. This perceived lack of organisational commitment also shaped this facilitator’s engagement with study delivery; describing not actively making time or seeking patients and suggesting that staff buy-in depended on the programme being formally launched and time being allocated for delivery. Comparison of accounts suggested that non-delivery was not consistently associated with lower confidence of preparedness. Opportunities to translate training into delivery appeared to depend more on how HF rehabilitation was organised and resourced locally, including service capacity, delivery responsibility and access to eligible patients.

A commonly reported challenge was the length of time between completing training and first delivery. For many facilitators, delays due to recruitment difficulties or service pressures meant that delivery occurred weeks or months after training. This affected recall and confidence. Several facilitators suggested that refresher input would have been beneficial in supporting the transition into delivery.

Yeah, it would be beneficial (…) if we’d have like a refresher course*.* Maya, delivered.

Facilitators also described the need for greater structure to support delivery, particularly when working independently or delivering sessions remotely. While the training clarified the overall components of REACH-HF, some facilitators reported difficulty maintaining structure across sessions in practice, especially where there were extended gaps between contacts. In response, facilitators described developing informal systems to support continuity.

The one big thing that I really struggled with was that I had no sort of paperwork framework. I didn’t have anything that was specifically for an initial assessment or specifically for the next assessment. Matthew, delivered.

Delivery experiences also highlighted selective enactment of programme components. For example, exercise specialists felt more comfortable delivering the exercise elements of REACH-HF. These decisions were framed as pragmatic responses to time constraints, patient needs and facilitators’ own professional backgrounds, rather than as deliberate departures from the programme.

Despite these challenges, facilitators described learning through delivery as an important source of growing confidence. Several noted that aspects of the training became more meaningful once applied in practice, and that confidence developed iteratively rather than being fully established at the point of training completion.

It’s not until you’re in the situation with the patient that I feel like you’ve fully appreciated it and fully learn Anna, delivered.

Translating REACH-HF into practice therefore involved ongoing calibration between training intentions, service realities and facilitators’ evolving confidence, with delivery itself becoming a key mode of learning rather than a straightforward enactment of the training.

## Discussion

This study explored whether a digitally delivered training programme could support quality REACH-HF delivery while addressing scalability constraints within current CR services. Fidelity findings suggested adequate enactment of most intervention components following digital training. Delivery was strongest for patient involvement, the physical activity programme and programme closure; needs assessment, tailored planning, HF understanding and progress monitoring were competent. Delivery of emotional support, medication-related discussion and exploration of caregiver-involvement was weaker and more variable. Caregiver emotion and well-being could not be meaningfully assessed because only one recorded case involved a caregiver. The qualitative findings contextualised these patterns, suggesting that intervention delivery was shaped not only by exposure to training but also by how facilitators engaged with, understood and consolidated training content within routine practice. Together, the findings align with conceptualisations of fidelity that distinguish between intervention training and intervention delivery,^29^ suggesting that variability in delivery may originate upstream in how training is operationalised and reinforced in practice.

The relative pattern of stronger and weaker domains descriptively resembled that reported in previous REACH-HF fidelity following 3-day face-to-face training.^10 30^ However, the small and uneven recording sample permits only historical contextualisation, not inference that digital and face-to-face training formats produced equivalent delivery. The relative difficulty observed in delivering some intervention domains may nevertheless reflect wider challenges associated with delivering complex, person-centred interventions in routine clinical settings rather than limitations specific to digital training. Similar patterns have been reported in other complex intervention and self-management studies, where delivery of psychosocial and caregiver-focused components appears particularly sensitive to practitioner experience, intervention complexity and local service conditions.^31 32^ In the present study, facilitators more readily incorporated components aligned with existing professional roles and established CR practices, while less familiar domains often required greater confidence, experience and opportunities for rehearsal and reflection in practice.

Facilitator recruitment and training completion were high, with 15 HCPs recruited and 14 completing both the eLearning and live training components. However, substantial delays in the time required to secure local governance approvals and activate participating sites meant that the patient recruitment and implementation window was significantly reduced. Moreover, due to the variation in time taken in securing approvals and site activation, the training delivery was staggered in cohorts. This meant that those trained in later cohorts had an even shorter period of time to recruit eligible patients and deliver the 12-week intervention. The principal implementation bottleneck therefore manifested after training, in the progression from training to patient recruitment and delivery but was partly produced by delays occurring earlier in study set up. Consequently only seven of the 14 HCPs who completed the training delivered REACH-HF during the available study period. Workforce disruption, limited service capacity, alternative CR provision preferences and underdeveloped HF referral pathways further constrained opportunities to initiate delivery. Among facilitators who remained available and contactable, non-delivery was not consistently associated with lower confidence or preparedness; some felt capable of delivering REACH-HF but lacked the operational opportunity, role ownership or access to eligible patients needed to do so. The progression rate should therefore be interpreted as evidence of a post-training implementation bottleneck within a constrained and uneven implementation period rather than as attrition attributable primarily to the digital training. These findings distinguish the potential scalability of facilitator training from that of REACH-HF delivery. The digital format may expand training capacity by reducing synchronous trainer time and enabling flexible, asynchronous participation. However, scalable intervention delivery also depends on services converting training into operational opportunities through protected learning time, clear delivery responsibilities, workable referral pathways, access to eligible patients and timely opportunities to begin delivery. This is consistent with previous REACH-HF implementation research identifying resources, commissioning and integration with HF services as important to adoption.^27^ Longer follow-up would be required to determine the extent to which trained facilitators subsequently delivered REACH-HF as part of routine practice.

Facilitators described protected training and intervention delivery time as vulnerable to displacement by routine clinical care pressures. Workforce shortages, competing clinical demands and COVID-19-related service pressures meant that scheduled learning time was often interrupted, rescheduled or absorbed into clinical work by service managers attempting to manage operational pressures. This contributed to fragmented engagement with asynchronous content and, for prioritising course over reflection, consolidation and rehearsal. Flexibility in digital training therefore does not remove the need for organisational investment in protected learning time. The findings support a blended approach in which asynchronous learning is complemented by synchronous discussion and skills practices, refresher support and practical scaffolding tools such as structured session guides and early opportunities to apply learning in practice. This is particularly relevant as HCP continuing professional development increasingly adopts flexible and self-directed digital formats.^33–35^ Assuming that digital training can be absorbed informally into existing workloads risks undermining both learning and subsequent intervention enactment.

A key strength of this study was the integration of competency-based fidelity assessment with qualitative exploration of facilitator experiences, enabling examination of both relative patterns in intervention delivery quality and the processes shaping enactment in practice. The inclusion in interviews of trained HCPs who did and did not progress to delivery provided insight into the transition from training into early delivery, a phase often under-examined in evaluations of workforce training. Several limitations should also be considered. Fidelity assessment was based on a small number of patients and recordings, with limited opportunities to observe facilitators repeatedly across the programme. The mean interval of approximately 4 months between training completion and first delivery may also have affected facilitators’ retention and application of delivery skills. Interview accounts supported this possibility: facilitators described extended delays as affecting recall and confidence and that refresher input would have supported the transition into delivery. However, fidelity data were available for only six facilitators and were unevenly clustered within facilitator-patient pairings, precluding any meaningful examination of whether longer delays were associated with systematically different fidelity profiles.

The fidelity sample may also have been affected by selection and reactivity biases. Only facilitators who delivered REACH-HF and returned usable recordings were represented, and their awareness of being recorded may have influenced how they delivered the intervention. Similarly, interview accounts may have been influenced by social desirability because the interviewer was a member of the research team. However, participants described both positive and substantive challenges relating to the training and its implementation, suggesting that their accounts were not uniformly favourable. Both fidelity assessors had been involved in the development and evaluation of REACH-HF and were not formally blinded to facilitator identity, site or session sequence, potentially introducing assessor bias. Although the double-coded recordings were scored independently and pre-consensus agreement was substantial, this does not exclude the possibility of shared bias. Finally, recording coverage varied substantially between facilitator-patient pairings, from one to eight sessions. Aggregating scores within pairings ensured that each pairing contributed equally to the item-level summaries, but estimates based on one or few recordings remained more susceptible to session-specific variation. The summary statistics should therefore be interpreted as descriptive patterns of delivery across intervention domains within this specific NHS implementation context rather than precise estimates of facilitator competence or delivery quality beyond this context.

### Conclusion

This study suggests that co-designed digital training may support HCPs to prepare for REACH-HF delivery. Adequate fidelity was observed across most assessable intervention components. However, further research with larger and more complete fidelity datasets is needed before firm conclusions can be drawn about overall delivery fidelity or equivalence with face-to-face training. HCPs generally perceived the training as usable and valuable, but their engagement and preparedness were shaped by prior experience, protected learning time and opportunities to apply their learning in practice. Confidence was consolidated through real-world delivery rather than training, training completion alone, highlighting the importance of timely delivery opportunities and ongoing practical and organisational support.

## Supplementary material

10.1136/bmjopen-2026-122821 online supplemental file 1

## Data Availability

No data are available. All data relevant to the study are included in the article or uploaded as supplementary information.
