# Supplementary material for: Digital training to support delivery of an evidence-based cardiac rehabilitation programme for heart failure: a mixed-methods evaluation of healthcare professionals’ training experiences and intervention fidelity
Source: BMJ Open. 2026 Oct 20;16(10):e122821. doi: 10.1136/bmjopen-2026-122821 (PMC13684163; doi:10.1136/bmjopen-2026-122821)
Supplement: Supplementary file 1 [file bmjopen-2026-122821.reviewer_comments.pdf]

PEER REVIEW HISTORY

BMJ Open publishes all reviews undertaken for accepted manuscripts. Reviewers are asked to complete a checklist review form and are provided with free text boxes to elaborate on their assessment. These free text comments are reproduced below.

ARTICLE DETAILS

Title (Provisional)

Digital training to support delivery of an evidence-based cardiac rehabilitation programme for heart failure: a mixed-methods evaluation of healthcare professionals' experiences and intervention fidelity

Authors

van Beurden, Samantha B. Barbara; Cross, Rosina; McDonagh, Sinead TJ; Deighan, Carolyn; Taylor, Rod; Doherty, Patrick Joseph; Ludman, Andrew; Clark, Liz; Dalal, Hasnain M; Greaves, Colin J

VERSION 1 - REVIEW

**Reviewer**	**1**
**Name**	**Okumura, Takahiro**
**Affiliation**	**Nagoya University Graduate School of Medicine Faculty of Medicine Department of Cardiology, Cardiology**
**Date**	**21-Jun-2026**
**COI**	**None**

In the manuscript entitled "Digital training to support scalable delivery of evidence-based rehabilitation for heart failure (REACH-HF): a mixed-method evaluation of facilitator training and intervention fidelity", the authors evaluated a co-designed digital facilitator training programme for REACH-HF using fidelity assessment of audio-recorded sessions and semi-structured interviews with healthcare professionals. These findings are scientifically interesting and have practical implications for scalable cardiac rehabilitation delivery. However, this manuscript has some concerns to be solved.

The number of recordings per patient varied considerably (mean 2.8, range 1–8), which may have introduced instability in fidelity scores. The authors are encouraged to discuss how this variability may have affected the reliability of item-level scores.

Key details of the digital training programme, including total learning hours, module structure, and completion rates, are not reported in the main text. Providing these details is needed for assessing the reproducibility and scalability of the training, which are central aims of this study.

The present study and the prior process evaluation (Frost et al., 2019) differ substantially in patient and facilitator characteristics, timing, and number of recordings.

While Table 2 presents fidelity scores alongside those from Frost et al., no statistical basis for this comparison is provided.

Only 7 of 14 trained HCPs delivered the intervention, yet no comparison of characteristics between those who did and did not deliver REACH-HF is presented.

Fidelity assessment was limited to facilitators who returned usable recordings, raising the possibility of selection and reactivity biases.

As the interviewer was a member of the research team, social desirability bias may have influenced participants' responses.

**Reviewer**	**2**
**Name**	**Jo, Sang-Ho**
**Affiliation**	**Hallym University Sacred Heart Hospital**
**Date**	**23-Jun-2026**
**COI**	**None**

MS No: BMJ Open : bmjopen-2026-122821

This manuscript addresses a relevant implementation question: whether digitally delivered facilitator training can support scalable delivery of the evidence-based REACH-HF cardiac rehabilitation program for patients with heart failure. The mixed-method design combining competency-based fidelity assessment with reflexive thematic analysis is appropriate in principle. However, the dataset is too small and too attenuated to bear the weight of the paper's central comparative claims. Several issues should be addressed, and the main contribution reframed accordingly. Major revision is required.

Major Comments

1. The fidelity comparison with Frost et al. (2019) is overinterpreted. Seventeen usable recordings from six facilitators -with a mean of 2.8 sessions per patient - cannot be benchmarked against the original REACH-HF process evaluation, which was conducted at substantially greater scale. Sparse and uneven session-level data in the present study are likely to reflect episodic variability rather than stable facilitator competence. The term "benchmarks" implies methodological equivalence that is not present here. The abstract, discussion, and conclusion should be revised to frame this explicitly as a descriptive, hypothesis-generating observation, with a clear statement that no formal statistical or non-inferiority comparison with face-to-face training was made or was possible.

2. The attrition from training to delivery is an underanalyzed implementation finding. Fourteen facilitators completed the digital training, but only seven delivered the intervention

-a delivery rate of 50% -and only six contributed usable fidelity data, corresponding to an evaluable fidelity-data rate of approximately 43%. This is not a minor logistical issue; it directly challenges the scalability argument. Four non-delivering facilitators participated in interviews, and their accounts represent an analytically distinct sub-group whose barriers - service capacity, role fit, confidence, protected time, organizational support - are central to the paper's stated research question. This opportunity was not taken. The Discussion must engage substantively with what this attrition means for real-world scalability.

3. No patient-level clinical outcome data are reported. Fidelity is an intermediate process measure; it is only meaningful to the extent it connects to patient outcomes. Eight patients were enrolled in the program, yet no patient-level data on quality of life, program completion, hospitalization, adverse events, or patient satisfaction are presented. The authors should state explicitly whether outcome data were collected. If they were not, all clinical implications must be correspondingly softened. If they were, their omission requires clear justification.

4. Fidelity scoring lacks sufficient methodological transparency. The two raters were both familiar with REACH-HF and involved in the study. It is not stated whether they were blinded to facilitator identity, site, delivery sequence, or time elapsed since training. Inter-rater reliability prior to consensus discussion should be reported - preferably as an intraclass correlation coefficient or weighted kappa. Without this, assessor bias and measurement imprecision cannot be adequately evaluated.

5. No sample size rationale is provided for either component of the study. For the fidelity assessment, there is no justification for the target number of facilitators, sessions, or patients. For the qualitative component, the authors should provide a clearer rationale for sample adequacy and information power, rather than relying only on descriptive reporting of the interview sample.

6. The application of Normalisation Process Theory adds limited analytical value. The three qualitative themes map almost directly onto NPT constructs, but the framework generates no mechanistic insight beyond what the inductive analysis already yields. NPT is used for labelling, not explanation. If it is to be retained, it should be applied to identify which specific collective action mechanisms were absent or deficient, and what this implies for implementation design — not simply to redescribe facilitators' experiences in theoretical vocabulary.

Minor Comments

1. The abstract's claim that delivery patterns were "broadly comparable" to face-to-face training should be removed or substantially softened.

2. The four-month mean gap between training completion and first delivery is a potential determinant of fidelity decay. Whether earlier delivery was associated with systematically different fidelity profiles deserves at least a brief descriptive note.

3. Table 2 should report standard deviations or interquartile ranges alongside means. Minima and maxima alone are not informative in a sample of this size.

4. Caregiver-related fidelity domains (items 10–11) are not interpretable, as only one recorded case included caregiver participation. This limitation should be stated clearly in the Results rather than left to a footnote in Table 2.

5. Three errors in the Supplementary File and manuscript require correction: (a) checklist items 4 and 5a share near-identical text and appear to contain a copy-paste error; (b) page 4 of the Introduction contains the typographical error "benchmarks based established"; (c) the StaRI checklist header refers to "Open Heart" rather than BMJ Open.

VERSION 1 - AUTHOR RESPONSE

Reviewer: 1
Dr. Takahiro Okumura, Nagoya University Graduate School of Medicine Faculty of Medicine Department of Cardiology
Comments to the Author:
In the manuscript entitled "Digital training to support scalable delivery of evidence-based rehabilitation for heart failure (REACH-HF): a mixed-method evaluation of facilitator training and intervention fidelity", the authors evaluated a co-designed digital facilitator training programme for REACH-HF using fidelity assessment of audio-recorded sessions and semi-structured interviews with healthcare professionals. These findings are scientifically interesting and have practical implications for scalable cardiac rehabilitation delivery. However, this manuscript has some concerns to be solved.

1.1 The number of recordings per patient varied considerably (mean 2.8, range 1–8), which may have introduced instability in fidelity scores. The authors are encouraged to discuss how this variability may have affected the reliability of item-level scores.

Thank you. We agree that the unequal number of recordings per facilitator-patient pairing may have affected the stability of the item-level fidelity scores. Scores were aggregated within facilitator-patient pairings so that each pairing contributed equally to the item-level summaries, irrespective of the number of recordings returned. Although this prevented pairings with more recordings from disproportionately influencing the findings, estimates based on single or few recordings remain more susceptible to session-specific variation than estimates based on several recordings. We have clarified the aggregation procedure in the Methods, reported the distribution of recordings in the Results, and expanded the limitations to emphasise that the item-level summaries represent descriptive patterns rather than precise estimates of stable facilitator competence.

1.2. Key details of the digital training programme, including total learning hours, module structure, and completion rates, are not reported in the main text. Providing these details is needed for assessing the reproducibility and scalability of the training, which are central aims of this study.

Thank you. We have revised the Methods to clarify the training structure, including the core modules, their sequence, and progression requirements, and the relationship between the eLearning and live session. We have also added the anticipated training duration: approximately 2 days in total, comprising of around 1.5 days of self-paced learning and a 3-hr live session. The Results clarify that

14 of the 15 recruited healthcare professionals completed the course, one withdrew during the online component due to long-term illness.

1.3. The present study and the prior process evaluation (Frost et al., 2019) differ substantially in patient and facilitator characteristics, timing, and number of recordings.

Thank you. We agree with the reviewer, and this relates to the reviewer's own additional concern 1.4 and reviewer 2's concern highlighted in 2.1. We have therefore prepared a comprehensive response to all, presented in 2.1.

1.4. While Table 2 presents fidelity scores alongside those from Frost et al., no statistical basis for this comparison is provided.

Thank you. The fidelity scores from Frost et al. have been removed from this table. See also our response to Reviewer comment 2.1.

1.5. Only 7 of 14 trained HCPs delivered the intervention, yet no comparison of characteristics between those who did and did not deliver REACH-HF is presented.

Thank you. Demographic questionnaire data were available for only eight interviewed facilitators (six who delivered REACH-HF and two who did not). Given the small, incomplete and imbalanced groups, a comparison of characteristics by delivery status would be potentially misleading. We have clarified this limitation in the manuscript. Instead, we compared the qualitative accounts of delivering and non-delivering facilitators, identifying differences in operational opportunities available to translate training into delivery rather than a consistent divide in confidence or preparedness (also in response to Reviewer comment 2.2).

1.6. Fidelity assessment was limited to facilitators who returned usable recordings, raising the possibility of selection and reactivity biases.

Thank you. We agree that fidelity could be assessed only for facilitators who progressed to delivery and for sessions with usable recordings, creating the potential for selection bias if those represented differed from those not represented. Facilitator's awareness of being recorded may also have influenced how they delivered the intervention. We have acknowledged these potential selection and reactivity biases in the study limitations.

1.7. As the interviewer was a member of the research team, social desirability bias may have influenced participants' responses.

Thank you. We agree that participants' accounts may have been influenced by social desirability because the interviewer was a member of the research team. We have acknowledged this in the study limitations.

Reviewer: 2
Dr. Sang-Ho Jo, Hallym University Sacred Heart Hospital
Comments to the Author:MS No: BMJ Open : bmjopen-2026-122821
This manuscript addresses a relevant implementation question: whether digitally delivered facilitator training can support scalable delivery of the evidence-based REACH-HF cardiac rehabilitation

program for patients with heart failure. The mixed-method design combining competency-based fidelity assessment with reflexive thematic analysis is appropriate in principle. However, the dataset is too small and too attenuated to bear the weight of the paper's central comparative claims. Several issues should be addressed, and the main contribution reframed accordingly. Major revision is required.

Major Comments

2.1. The fidelity comparison with Frost et al. (2019) is overinterpreted. Seventeen usable recordings from six facilitators -with a mean of 2.8 sessions per patient - cannot be benchmarked against the original REACH-HF process evaluation, which was conducted at substantially greater scale. Sparse and uneven session-level data in the present study are likely to reflect episodic variability rather than stable facilitator competence. The term "benchmarks" implies methodological equivalence that is not present here. The abstract, discussion, and conclusion should be revised to frame this explicitly as a descriptive, hypothesis-generating observation, with a clear statement that no formal statistical or non-inferiority comparison with face-to-face training was made or was possible.

Thank you for this constructive comment. We agree that achieved fidelity dataset does not support benchmarking or inference of equivalence with the original REACH-HF process evaluation. This concern also relates to Reviewer 1's comments 1.3. and 1.4.

As explained in response to 2.5, we intended to generate a dataset broadly similar in scope to the original evaluation. Although a similar number of facilitators contributed recordings (six vs seven), the present dataset included substantially fewer patients and recordings per patient resulting in a large difference in volume of recordings (17 vs 110). Both datasets had uneven recording coverage, but the much smaller present dataset was more susceptible to patient- and session-specific variation and could not support a formal statistical, equivalence, or non-inferiority comparison. No such comparison was undertaken.

We have therefore removed the previous scores from Table 2 and removed "benchmark" terminology and comparative claims from the Abstract (Reviewer comment 2.7) and Conclusion. The original findings are now referenced only in the Discussion as historical context for the broad pattern of stronger and weaker delivery domains, with an explicit statement that this does not demonstrate equivalence between training formats. Further detail on the instability associated with uneven recording coverage is provided in response to Reviewer comment 1.1 and the additional reporting of within-study distributions in response to the Reviewer comment 2.9. The findings are now framed throughout as descriptive patterns within the present evaluation.

2.2. The attrition from training to delivery is an underanalyzed implementation finding. Fourteen facilitators completed the digital training, but only seven delivered the intervention -a delivery rate of 50% -and only six contributed usable fidelity data, corresponding to an evaluable fidelity-data rate of approximately 43%. This is not a minor logistical issue; it directly challenges the scalability argument. Four non-delivering facilitators participated in interviews, and their accounts represent an analytically distinct sub-group whose barriers - service capacity, role fit, confidence, protected time, organizational support - are central to the paper's stated research question. This opportunity was not taken. The Discussion must engage substantively with what this attrition means for real-world scalability.

We thank the reviewer for highlighting this missed analytical opportunity. Addressing it has added analytical depth and strengthened the paper's alignment with its stated objectives. We have clarified

the participant flow, distinguishing post-training withdrawal or loss to follow-up (illness, redeployment, and inability to contact) from non-progression to delivery among facilitators who remained in the study (e.g., limited service capacity, unclear delivery roles and difficulties recruiting eligible patients). We have also identified quotations by delivery status, explicitly compared delivering and non-delivering facilitators and revised the Discussion to distinguish the potential scalability of digital training from scalable intervention delivery, contextualising this interpretation within previous REACH-HF implementation research.

2.3. No patient-level clinical outcome data are reported. Fidelity is an intermediate process measure; it is only meaningful to the extent it connects to patient outcomes. Eight patients were enrolled in the program, yet no patient-level data on quality of life, program completion, hospitalization, adverse events, or patient satisfaction are presented. The authors should state explicitly whether outcome data were collected. If they were not, all clinical implications must be correspondingly softened. If they were, their omission requires clear justification.

Thank you. Patient-level clinical outcomes were not collected because this study focused on facilitator training and fidelity of intervention delivery rather than clinical effectiveness or associations between fidelity and patient outcomes. We have clarified this in the Methods.

In addressing related reviewer comments, particularly 2.1, 2.2, and 2.7, we have also revised the Abstract, Discussion, and Conclusion to frame the fidelity findings descriptively, remove terminology which could be perceived as implying comparative claims, and distinguish between the potential scalability of facilitator training from scalable intervention. We believe these revisions have helped to clarify, and in some cases better align, our interpretations with what the study can support, without implying patient benefit or clinical effectiveness. References to the clinical effectiveness of REACH-HF in the Introduction relate to previous published trial evidence.

2.4. Fidelity scoring lacks sufficient methodological transparency. The two raters were both familiar with REACH-HF and involved in the study. It is not stated whether they were blinded to facilitator identity, site, delivery sequence, or time elapsed since training. Inter-rater reliability prior to consensus discussion should be reported - preferably as an intraclass correlation coefficient or weighted kappa. Without this, assessor bias and measurement imprecision cannot be adequately evaluated.

Thank you. We agree that the original manuscript did not provide sufficient information about the fidelity-scoring process or the potential for assessor bias. We have expanded the Methods section to describe the raters' expertise, absence of formal blinding, independence of the initial scoring, criteria for concordance, and the consensus procedure. We also clarify that inter-rater reliability was calculated using the original pre-consensus ratings. Quadratic-weighted Cohen's kappa was selected because the competency scale produces order ratings, including half-point scores.

The Results now clarify that 13 of the 17 recordings were independently scored by both raters and explain how and why the remaining four were assessed by one rater. We report substantial pre-consensus agreement. Finally, we acknowledge in the Discussion that the raters' involvement in REACH-HF and lack of formal blinding may have introduced assessor bias, and that independent scoring and substantial agreement do not exclude the possibility of shared bias.

2.5. No sample size rationale is provided for either component of the study. For the fidelity

assessment, there is no justification for the target number of facilitators, sessions, or patients. For the qualitative component, the authors should provide a clearer rationale for sample adequacy and information power, rather than relying only on descriptive reporting of the interview sample.

Thank you. We have added a target sample size and sample adequacy rationales.

Our intention had been to generate a dataset broadly similar in scope with the REACH-HF process evaluation (Frost et al. 2019) which had been nested in a multi-site randomised controlled trial with four sites (Dalal et al. 2019). Therefore, for the fidelity assessment, we targeted facilitators across five cardiac rehabilitation services, with three per service, to provide breadth across service contexts and within-service depth while allowing for attrition. Each facilitator could deliver REACH-HF to up to three patients, providing a maximum of 45 facilitator-patient pairings and approximately 360-540 potentially recordable sessions. Based on the 34% recording availability achieved in the original REACH-HF process evaluation, we anticipated approximately 122-184 recordings (vs the 110 recordings from seven facilitators in the REACH-HF process evaluation). This has been added to the Method section.

For the qualitative component, all trained facilitators who remained available and contactable were interviewed (n=11), including seven who delivered REACH-HF and four who did not. We have clarified in the Results that the achieved sample provided adequate information power because it addressed a focused experience (digital REACH-HF training and its translation into delivery) within a highly specific sample (all HCPs who had completed the training), included variation in delivery status, professional roles, and service context, and generated detailed accounts supporting comparison across these experience.

2.6. The application of Normalisation Process Theory adds limited analytical value. The three qualitative themes map almost directly onto NPT constructs, but the framework generates no mechanistic insight beyond what the inductive analysis already yields. NPT is used for labelling, not explanation. If it is to be retained, it should be applied to identify which specific collective action mechanisms were absent or deficient, and what this implies for implementation design — not simply to redescribe facilitators' experiences in theoretical vocabulary.

Thank you. We initially retained Normalisation Process Theory (NPT) as a secondary interpretive framework to connect our data-led explanations with established and widely recognised implementation concepts. However, we agree that its application did not add sufficient additional explanatory insight beyond what had resulted from our initial analysis. We have therefore removed NPT from the Methods, Results, and Discussion (and Supplementary File). This has also helped to offset the additional text required to address other reviewer comments and maintain the manuscript's focus and length.

Minor Comments

2.7. The abstract's claim that delivery patterns were "broadly comparable" to face-to-face training should be removed or substantially softened.

In addressing this Reviewer's earlier comment (2.1) we have removed this sentence.

2.8. The four-month mean gap between training completion and first delivery is a potential determinant of fidelity decay. Whether earlier delivery was associated with systematically different fidelity profiles deserves at least a brief descriptive note.

Thank you. We agree that the interval between training completion and first delivery is a plausible influence on the retention and application of intervention-delivery skills. However, fidelity data were available only for six facilitators, with recordings unevenly clustered within facilitator-patient pairings, and therefore did not support a meaningful analysis of the association between time to delivery and fidelity. We have not undertaken a formal association of subgroup analysis. Instead, we have revised the Discussion to acknowledging that these delays may have contributed to variation in fidelity and relate this possibility to the qualitative findings, in which facilitators described extended gaps as affecting their recall of the intervention and confidence to deliver it.

2.9. Table 2 should report standard deviations or interquartile ranges alongside means. Minima and maxima alone are not informative in a sample of this size.

Thank you. We have revised Table 2 to report the median and interquartile ranges (25th-75th percentiles) alongside the mean. To mitigate the increase in table size we have merged the minimum and maximum fidelity scores into range. The IQRs were calculated across the six facilitator-patient-level aggregated scores rather than across the 17 individual recordings (See also Reviewer comment 1.1), consistent with the unit of analysis. IQRs were not calculated for Items 10 and 11 because only one recorded case involved caregiver participation. We have reported this in the footnote of the table.

2.10 Caregiver-related fidelity domains (items 10–11) are not interpretable, as only one recorded case included caregiver participation. This limitation should be stated clearly in the Results rather than left to a footnote in Table 2.

Thank you. We have expanded the Results to state explicitly that Items 10 and 11 cannot be meaningfully interpreted because they required a caregiver to be present, which occurred in only one recorded case. We also clarified that Item 9 remained applicable to all sessions, because it included assessment of whether facilitators explored and encouraged the involvement of a caregiver or other supporter, regardless of whether one was present. Its lower score therefore reflects inconsistent exploration of potential caregiver involvement rather than the limited number of caregivers attending sessions.

2.11 Three errors in the Supplementary File and manuscript require correction: (a) checklist items 4 and 5a share near-identical text and appear to contain a copy-paste error; (b) page 4 of the Introduction contains the typographical error "benchmarks based established"; (c) the StaRI checklist header refers to "Open Heart" rather than BMJ Open.

Thank you for identifying these errors. (a) We corrected the copy-paste error by adding the appropriate content for Item 4, Building understanding of HF. Reviewing the checklist also identified two further omissions: the cross-cutting facilitation skills have now been added at the beginning, and Items 9 and item 12 have been updated to reflect the scoring refinements described in the manuscript. (b) The typographical error in the Introduction was corrected as part of the revisions addressing Reviewer comment 2.1. (c) We corrected the StaRI header to refer to BMJ Open. The reference to Open Heart was a legacy of the manuscript's transfer between the two BMJ journals.

Reviewer: 1

If you have selected 'Yes' above, please provide details of any competing interests.: Not applicable

Reviewer: 2
If you have selected 'Yes' above, please provide details of any competing interests.: N/A

VERSION 2 - REVIEW

Reviewer **1**

Name **Okumura, Takahiro**

Affiliation **Nagoya University Graduate School of Medicine Faculty of Medicine Department of Cardiology, Cardiology**

Date **27-Sep-2026**

COI

The authors have adequately addressed my previous concerns. I have only a few minor comments.

#Several editorial errors remain, including a broken cross-reference in the Results ("Error! Reference source not found."), an incomplete sentence in the Abstract, and a duplicated row in Supplementary Table 1. A careful proofread of the entire manuscript is recommended.
