# Supplementary material for: Digital training to support delivery of an evidence-based cardiac rehabilitation programme for heart failure: a mixed-methods evaluation of healthcare professionals’ training experiences and intervention fidelity
Source: BMJ Open. 2026 Oct 20;16(10):e122821. doi: 10.1136/bmjopen-2026-122821 (PMC13684163; doi:10.1136/bmjopen-2026-122821)
Supplement: Supplementary file 2 [file bmjopen-16-10-s001.docx]

**Dr Samantha B van Beurden, Dr Rosina Cross, Dr Sinead TJ McDonagh, Professor Rod S Taylor, Professor Patrick J Doherty, Dr Andrew Ludman, Liz Clark, Dr Hasnain Dalal, and Professor Colin J Greaves**

Press Ctrl+Home to get back to this page.

[Co-Design Process	2](#_Toc225873150)

[Topic Guide	3](#_Toc225873151)

[Fidelity checklist	5](#_Toc225873152)

[Expanded Qualitative Analytic Methods	7](#_Toc225873153)

# Co-Design Process

Co-design partners were recruited through social media (X, Facebook, and LinkedIn) and existing Rehabilitation Enablement in Chronic Heart Failure (REACH-HF) networks of cardiac rehabilitation (CR) staff trained in the intervention. Partners included patients with heart failure (HF) and informal caregivers (n=7), healthcare professionals (HCPs) involved in CR or HF care (n=10), members of the original REACH-HF intervention development team (n=3), and REACH-HF facilitator training providers (n=3).

The co-design process took place between May 2021 and March 2022 and involved five iterative workshops: three with patients and caregivers, and two with healthcare professionals and training providers. Workshops were facilitated by members of the research team (RC, SvB, SMcD), who had experience in participatory methods and digital intervention design. Additional input was gathered through one-to-one consultations and asynchronous feedback for contributors unable to attend the workshops.

Co-design activities focused on four areas: (1) confirming and refining the intended learning outcomes for the digital course, to ensure alignment with the knowledge, skills, and facilitation capabilities required for REACH-HF delivery; (2) reviewing the structure, content, and materials of the existing paper-based, synchronously delivered training; (3) identifying barriers to engagement and scalability; and (4) co-creating a usable, engaging, and contextually appropriate digital learning experience.

A structured wireframing process was used to support collaborative decisions about course structure, sequencing, navigation, tone, and key platform features. This helped translate the existing training into a digital format while retaining its emphasis on person-centred facilitation and applied learning. The platform was then developed by Health and Care Innovations (HCI) in collaboration with the research team, informed by this co-design input.

Co-design partners also contributed to the development of scenario-based videos, case studies, interactive exercises, reflection prompts, and quiz-based knowledge checks. These features were intended to support active learning and reflect the relational and facilitation skills needed to deliver REACH-HF effectively in practice. Course progression criteria were also co-defined to guide learners through the self-paced modules, and a live 3-hour trainer-led session was incorporated to provide opportunities for discussion, skills practice, and feedback.

Ideas and feedback generated through workshops and consultations were documented after each stage, reviewed by the core research team, and used to iteratively refine the training content, format, and user experience.

# Topic Guide

## Experiences of being recruited to the REACH-HF Digital Training Programme

Could you tell me about how you were first approached by your line manager to take part in the REACH-HF training? (Prompt: Were time commitments made clear, was time scheduled into your work calendar, did you feel like you were supported sufficiently to participate in the training).

## Experiences of delivering the REACH-HF programme to patients

Can you tell me what, if anything, worked particularly well during your time delivering the REACH-HF programme?

Which, if any, aspects of the programme were more challenging to deliver than others?

- How, if at all, were these overcome?

Which aspects of the programme, if any, didn’t work well?

- How would you address these aspects for future delivery, if at all?

What were some of the challenges, if any, in getting your local service set up to deliver REACH-HF? *E.g. referral processes; any impact on the wider team?*

## Moving on to your experiences of the REACH-HF training programme you took part in.

Thinking back to the training course, what were your initial thoughts when you first started exploring the website?

How easy or hard was it to use the online training course?

- Did you experience any technical barriers? (If so, where, what, and what impact?)
- Did you experience any language barriers? (If so, where, what, and what impact?)
- What were the instructions like? (e.g., clarity, amount)
- How did you find navigating the website?

What was your impression of the training content?

Was there anything missing or that could have been expanded on more to prepare you better for real-world delivery?

What aspects of the training did you find most useful?

- Which aspects of the training were less useful or hard to engage with? If so, what?

When you completed the digital training how confident were you, if at all, that you could deliver the REACH-HF programme?

- If not, what were you less confident about? Did this remain the same or change over time?
- If so, what helped you to improve your confidence in delivering this aspect of the programme?

In what ways, if any, could the training be improved?

- Understanding of heart failure
- Physical activity
- Stress and anxiety
- Self-management
- Long-term support
- Engaging the care giver
- Setting up the programme in your local CR service

# Fidelity checklist

For all items: Person centred counselling techniques using OARs (Open questions, Affirmation, Reflective listening, Summaries) and Ask-Tell-Discuss techniques for assessment and to exchange information to identify barriers and explore ways to overcome them.

1. **Active patient involvement**

Encourage the participant to be actively involved in the consultation, maximise the patient’s autonomy by developing intrinsic motivation (by setting goals and actions important to and enjoyable for the patient) and encouraging the patient to come up with ideas for improving the situation. However, the consultation should be guided and stay on topic. Overall, the patient should be increasingly empowered to take control of self-care. Interactions should be encouraging, respectful, and non-judgemental (as opposed to a didactic, telling or persuading style).

2. **Assessing the patient’s current situation and needs**

Work with the patient to assess their current situation. Seek to **identify** ALL of the following over the first 1-2 sessions: Identify (1) their current understanding of Heart Failure and address misconceptions, (2) discuss the most important issue currently for the patient, (3) how well are they monitoring their fluids, (4) how appropriately are they using medications, (5) is there any obvious immediate clinical need, (6) how much stress or anxiety do they have, (7) how much physical activity are they doing and at what intensity, and (8) what other concerns or questions they may have.

3. **Formulating an appropriate (individualised) treatment plan**

Work with the patient to formulate an appropriate treatment plan based on the patient’s current situation. This should aim to **address** (as a minimum) **ALL** of the 7 or 8 items in Point 2. The treatment plan will be staged over time, aiming to work on a few topics initially and introducing other elements as the programme continues. It is **best practice** to summarise the treatment plan at the end of the session “what we have said today is …”.

4. **Building Understanding of Heart Failure**

Elicit the patient’s current understanding of heart failure and seek to build their ‘illness model’ in terms of understanding the Identity, Causes, Consequences, Cure /control options and Timeline associated with the condition. This process may take several weeks and should be reinforced as the programme progresses.

5. **A - Supporting Self-monitoring and Progress Tracking**

Agree a verbal plan of action for the following week(s) with the patient and discuss the use of the Progress Tracker (and other tools) to keep track of progress and as a way of recording any problems in completing the activities and any benefits that might be associated with the planned activities.

5. **B - Reviewing progress and problem-solving**

Work with the patient to review progress with all planned changes and with achieving the targets set out in the action plan. Celebrate and reinforce and reflect on any successes. Discuss any setbacks with the patient and work together to revise the plans where necessary. In relation to Point 7 – randomly check if patient is still exercising at the appropriate intensity by asking them to complete an exercise at their current target level. Revise target level if appropriate (ie. Go up a level if the patient is not working at moderate intensity, they’re fitness has increased so they need to work harder/do more to achieve moderate intensity).

6. **Make a specific action plan for physical activity, based on the activities selected by the patient**

Using the template in the HF manual, work with the patient to agree a written or verbal plan of action for engaging in one of the exercise options over the following week(s). This should include discussion of breathing and co-completion of an exercise or walk to help the patient to recognise the **appropriate intensity (moderate)** they should be working at, to ensure that this is achieved for any activity included in the action plan.

7. **Addressing emotional consequences of heart failure**

Help the patient to recognise and address any significant stress, anxiety, anger, depression or other negative feelings that are related to having heart failure. Seek to normalise such feelings and help the patient to access and work through relevant sections of the manual. If these problems are severe or prolonged facilitate a referral to relevant care services.

8. **Addressing medication issues**

Help the patient to recognise and address any significant problems or concerns relating to their medications. Help the patient to work through relevant sections of the manual. This might include (1) problems in organising /taking the medications, (2) knowing what to do if they get a cold or forget a dose, (3) identifying possible side effects and seeking help to minimise them, (4) avoiding over-the-counter medications. For some patients, it may include discussing self-titration of diuretics (water tablets) in response to symptoms /swelling (using the Traffic Light plan as a guide).

9. **Supporter (caregiver) involvement (applicable to all sessions)**

Actively explore and encourage engagement of supporter or caregiver as a co-facilitator of the intervention. Where a caregiver is present/engaged: Tailor the intervention to work with the caregiver’s abilities and availability to provide support to the cared for person with self-management of their heart failure. Provide the Family & Friends Resource, a brief overview of what it contains, and identify some key sections for the caregiver to read.

10. **Addressing emotional consequences of being a supporter/caregiver (as applicable)**

Help the caregiver to recognise and address any significant stress, anxiety, anger, depression or other negative feelings that are related to becoming a caregiver and supporting someone with heart failure. Seek to normalise such feelings and help the caregiver to access and work through relevant sections of the Family & Friends Resource. This includes facilitating a referral for a carer’s assessment if the caregiver wishes, plus referral to other relevant care services as appropriate.

11. **Caregiver health and wellbeing (as applicable)**

Help the caregiver to prioritise and look after their own health and well-being.

12. **Bringing the programme to a close**

Inform, and remind, the patient (and caregiver) of when the programme would be/ is coming to an end. Progress should be consolidated and reinforced. Plans for long-term sustainability of activities and strategies learned for managing heart failure should be discussed. This includes (1) discussing the importance of maintaining these self-care behaviours, (2) working with the patient (caregiver) to identify or informing the patient (and caregiver) of any ongoing support networks available in the local area. In the final session, in addition to the above, discharging planning as appropriate to the local service.

Adapted from: Frost J, Wingham J, Britten N, et al. Home-based rehabilitation for heart failure with reduced ejection fraction: mixed methods process evaluation of the REACH-HF multicentre randomised controlled trial. *BMJ Open*; 9. Epub ahead of print 1 August 2019. DOI: [10.1136/bmjopen-2018-026039](https://doi.org/10.1136/bmjopen-2018-026039).

# Expanded Qualitative Analytic Methods

Our approach was informed by a pragmatic epistemology, recognising participants’ accounts as both reflections of lived experience and shaped by the organisational, relational, and structural contexts in which REACH-HF was delivered. Interview data were therefore treated as contextually situated and interpretive, while also offering insight into the processes and mechanisms influencing how facilitator training was received, understood, and enacted in practice.

Data were analysed using Reflexive Thematic Analysis (RTA)^1^, selected for its flexibility and its capacity to support a meaning-centred, interpretive analysis of how and why experiences occur within context. Analysis was primarily inductive, allowing themes to be generated from the data, while attending to both semantic and latent meanings.

Analysis was led by RC, with ongoing reflexive dialogue with SvB (both experienced qualitative researchers in the evaluation of complex interventions). Transcripts were read and re-read alongside fieldnotes to support familiarisation. Iterative coding involved the development and refinement of initial descriptive codes through constant comparison across transcripts, analytic memo writing, and team discussion. Coding captured both experiences of the training programme and subsequent intervention delivery, including where participants reflected on the translation of training into practice.

Codes were organised into candidate themes representing patterned meaning across the dataset, which were refined through iterative engagement with the full dataset to ensure coherence, internal consistency, and distinctiveness. NVivo software was used to support data management and organisation.

Throughout, analysis was underpinned by researcher reflexivity. Both analysts had prior involvement in the development and evaluation of Digital REACH-HF^2^, bringing contextual insight alongside the potential for pre-existing assumptions. Reflexive practices (including analytic discussion, reflective note-taking, and active searching for disconfirming cases) were used to critically examine interpretations and ensure they remained grounded in participants’ accounts.

Consistent with the epistemological foundations of RTA, we did not seek inter-rater reliability, formal saturation, or post hoc member checking, as these approaches assume a fixed or singular interpretation of qualitative data. Instead, rigour was achieved through depth of engagement, iterative analysis, reflexive dialogue, and transparent analytic decision-making. In-interview reflection was used to check understanding and support deeper exploration, and transferability was supported through detailed description of the study context, participants, and intervention.

Analysis and reporting were conducted in line with the Reflexive Thematic Analysis Reporting Guidelines (RTARG), with themes presented as interpretive accounts supported by illustrative quotations to convey both shared patterns and nuanced variation.

In the Results section, themes were presented as interpretive narratives supported by illustrative participant quotations to show how interpretations were grounded in the data. Quotations were selected to reflect both patterned meaning across the dataset and variation within themes, including typical, divergent, and more nuanced accounts. The presentation of findings aimed to move beyond descriptive summary by offering analytic commentary on the meanings and contextual influences shaping facilitators’ experiences of the training and its enactment in practice.

**Development of Themes: From Descriptive Categories to Interpretive Themes**

Initial coding and data organisation involved grouping material into descriptive categories that broadly reflected areas explored in the interview topic guide, such as enrolment into training, platform usability, training content, live sessions, and perceived outcomes of training and delivery. These categories provided a practical way of organising the dataset and informing interim reporting. However, they were not treated as the final analytic output.

Through iterative comparison across transcripts, reflexive discussion, and analytic memo-ing, these descriptive categories were progressively reworked into higher-order interpretive themes that captured patterned meaning across facilitators’ accounts and illuminated the processes shaping how training was understood, experienced, and translated into practice. The final themes therefore moved beyond topic-based description to provide an interpretive account of facilitators’ sense-making, capability development, and enactment of REACH-HF in routine service contexts. Table 1 provides an overview of how early descriptive categories contributed to the development of the final themes reported in the manuscript.

Table 1 Analytic progression from descriptive categories to final interpretive themes

| **Descriptive category** | **Key focus of descriptive category** | **Final interpretive theme (reported in Results)** |
| --- | --- | --- |
| Enrolment factors | How facilitators were approached, expectations set, perceived obligation versus opportunity | Theme 1: Making sense of REACH-HF and my role… |
| Motivations to engage and deliver REACH-HF | Willingness to engage, perceived legitimacy, professional fit, perceived value | Theme 1: Making sense of REACH-HF and my role… |
| Time allocated to training | Protected time, workload pressures, mismatch between expectations and reality | Theme 2: Developing capability and confidence through the training |
| Communications of training requirements | Clarity of expectations, understanding of training load, preparedness | Theme 2: Developing capability and confidence through the training |
| Perceived platform usability | Access, navigation, clarity of instructions, technical barriers | Theme 2: Developing capability and confidence through the training |
| Platform errors and technical issues | “Multiple Choice Question” glitches, frustration, need for technical support | Theme 2: Developing capability and confidence through the training |
| Perceived appropriateness of training content | Relevance, validation of existing practice, alignment with professional knowledge | Theme 1: Making sense of REACH-HF and my role |
| Extensiveness of content | Volume of material, pacing, perceived information overload | Theme 2: Developing capability and confidence through the training |
| Training videos and learning resources | Learning preferences, perceived realism of role play, engagement with materials | Theme 2: Developing capability and confidence through the training |
| Live training sessions | Interaction, opportunities for clarification, reassurance, credibility of trainers | Theme 2: Developing capability and confidence through the training |
| Live training sessions | Interaction, opportunities for clarification, reassurance, credibility of trainers | Theme 2: Developing capability and confidence through the training |
| Perceived confidence and readiness to deliver REACH-HF | Self-reported confidence, perceived readiness, reliance on prior role experience | Theme 3: Translating training into practice: … |
| REACH-HF session frameworks and tools | Need for structure, checklists, and scaffolding in practice | Theme 3: Translating training into practice: … |
| Extended training periods / refresher courses | Gaps between training and delivery, perceived decay of confidence over time | Theme 2: Developing capability and confidence through the training  Theme 3: Translating training into practice: … |
| Training outcomes and adaptations | Learning through delivery, adjustment of practice, use of workarounds | Theme 2: Developing capability and confidence through the training  Theme 3: Translating training into practice: … |

1.	Braun V, Clarke V. Reflecting on reflexive thematic analysis. *Qualitative Research in Sport, Exercise and Health* 2019; 11: 589–597.
<https://doi.org/10.1080/2159676X.2019.1628806>

2.	van Beurden SB, Cross R, McDonagh STJ, et al. Digital adaptation of the clinically effective REACH-HF home-based cardiac rehabilitation programme for people living with heart failure (D:REACH-HF). *DIGITAL HEALTH* 2025; 11: 20552076251406545. <https://doi.org/10.1177/205520762514065>
